# microRNA Expression in Ethnic Specific Early Stage Breast Cancer: an Integration and Comparative Analysis

**DOI:** 10.1038/s41598-017-16978-y

**Published:** 2017-12-04

**Authors:** Farah J. Nassar, Rabih Talhouk, Nathalie K. Zgheib, Arafat Tfayli, Maya El Sabban, Nagi S. El Saghir, Fouad Boulos, Mark N. Jabbour, Claude Chalala, Rose-Mary Boustany, Humam Kadara, Zhou Zhang, Yinan Zheng, Brian Joyce, Lifang Hou, Ali Bazarbachi, George Calin, Rihab Nasr

**Affiliations:** 1 Department of Biology, Faculty of Arts and Sciences, American University of Beirut, Beirut, Lebanon; 2 Department of Pharmacology and Toxicology, Faculty of Medicine, American University of Beirut, Beirut, Lebanon; 3 Department of Internal Medicine, Faculty of Medicine, American University of Beirut, Beirut, Lebanon; 4 Department of Biochemistry, Medical University of South Carolina, Charleston, South Carolina, USA; 5 Department of Pathology, Faculty of Medicine, American University of Beirut, Beirut, Lebanon; 6 Centre for Molecular Oncology, Barts Cancer Institute, Queen Mary University of London, Charterhouse Square, London, EC1M 6BQ UK; 7 Department of Pediatrics and Adolescent Medicine, American University of Beirut Medical Center, Beirut, Lebanon; 8 Department of Biochemistry and Molecular Genetics, American University of Beirut Medical Center, Beirut, Lebanon; 9 Duke University Medical Center, Durham, NC USA; 10 Center for Population Epigenetics, Department of Preventive Medicine, Feinberg School of Medicine, Northwestern University, Chicago, IL USA; 11 Driskill Graduate Program in Life Sciences, Northwestern University Feinberg School of Medicine, Chicago, IL USA; 12 Institute for Public Health and Medicine, Northwestern University Feinberg School of Medicine, Chicago, IL USA; 13 Division of Epidemiology/Biostatistics, School of Public Health, University of Illinois-Chicago, Chicago, IL USA; 14 Robert H. Lurie Comprehensive Cancer Center, Northwestern University Feinberg School of Medicine, Chicago, IL USA; 15 Department of Medicine, Center for Global Health, Northwestern University Feinberg School of Medicine, Chicago, IL USA; 16 Department of Experimental Therapeutics, Division of Cancer Medicine, University of Texas MD Anderson Cancer Center Houston, Texas, USA; 17 Department of Anatomy, Cell Biology and Physiological Sciences, Faculty of Medicine, American University of Beirut, Beirut, Lebanon

## Abstract

Breast cancer (BC) has a higher incidence in young Lebanese woman as compared to the West. We assessed the microRNA (miRNA) microarray profile of tissues derived from Lebanese patients with early BC and performed mRNA-miRNA integration analysis. 173 miRNAs were significantly dysregulated in 45 BC versus 17 normal adjacent breast tissues, including 74 with a fold change more than two of which 17 were never reported before in cancer. Integration analysis of mRNA-miRNA microarray data revealed a potential role of 51 dysregulated miRNA regulating 719 tumor suppressive or oncogenic mRNA associated with increased proliferation and decreased migration and invasion. We then performed a comparative miRNA microarray profile analysis of BC tissue between these 45 Lebanese and 197 matched American BC patients. Notably, Lebanese BC patients had 21 exclusively dysregulated miRNA (e.g. miR-31, 362-3p, and 663) and 4 miRNA with different expression manner compared to American patients (e.g. miR-1288-star and 324-3p). Some of these differences could reflect variation in patient age at diagnosis or ethnic variation affecting miRNA epigenetic regulation or sequence of miRNA precursors. Our data provide a basis for genetic/epigenetic investigations to explore the role of miRNA in early stage BC in young women, including ethnic specific differences.

## Introduction

Despite advances in detection and treatment methods, breast cancer still remains the number one cancer killer of women worldwide. In Lebanon, the age-standardized incidence rate of breast cancer is lower than that in developed countries but higher than in other Arab and Asian populations, with a notable increase among patients younger than 40 years old^1^. These young patients tend to have more aggressive cancer with worse prognosis and survival in spite of hormone and chemo-therapy^2^. Even though mammography is the gold standard diagnostic tool for breast cancer detection, its routine use is not recommended for women younger than 40 years old who are at risk for aggressive breast cancer in our population; thus research efforts have been focused on finding new biomarkers for breast cancer prevention and detection, particularly in ethnic specific populations.

microRNA (miRNA) are small non-coding 18–25 nucleotide RNA molecules currently being studied as potential diagnostic, prognostic and therapeutic biomarkers for cancer and other diseases. Extensive research on these post-transcriptional modulators has proven that they are deregulated in breast cancerous tissues and even in biological fluids from breast cancer patients^3,4^. miRNA can act as master players at any stage of breast cancer development by targeting multiple mRNA that are implicated in tumor suppressor or oncogenic signaling pathways^5^.

Recently, we have investigated the expression patterns of five candidate miRNAs (miR-10b, miR-148b, miR-221, miR-21, and miR-155) previously known to be dysregulated in breast cancer, in Lebanese breast cancer patients of various clinical and histopathological presentations^6^. This miRNA expression pilot study in Lebanese patients has revealed a different expression pattern for some miRNAs (miR-148b and miR-221) compared to what has been reported in the West. This suggests the need for an overall miRNA microarray profiling of tissues taken from Lebanese breast cancer patients along with a global mRNA profile analysis of the same tissues for mRNA-miRNA integration analysis and for comparative study of our miRNA profile to that of American specimens. Hence, the aim of this study is to examine the miRNA expression in ethnic specific breast cancer tissues and to predict their role in breast cancer development through miRNA-mRNA integration analysis.

## Results

### Patient Characteristics

The clinical and pathological data have been retrieved for all 62 samples of breast cancer patients used for the miRNA microarrays (Table 1). Pathologically, all cases were invasive ductal carcinoma (IDC) and estrogen receptor (ER) positive, 97.8% were progesterone receptor (PR) positive and 24.5% had human epidermal growth factor receptor 2 (HER2) over-expression. Most tumors were moderately differentiated (44.4% for tumor grade 2), 46.7% were with local growth and an average tumor size of two to five centimeters (T2) and 54.8% had lymph node involvement. None of the patients had distant metastases and were accordingly considered early stage breast cancer.

**Table 1:** Clinical and pathological characteristics of Lebanese breast cancer tissues used in microarray and RT-qPCR.

Patient Characteristics	Assay Type	
	Microarray	RT-qPCR
	N(%)	N(%)
Normal	17 (27.4)	10 (33.3)
Tumor	45 (72.6)	20 (66.7)
*Age*		
≤40 years	19 (42.2)	6 (30)
>40 years	26 (57.8)	14 (70)
*Menopausal status*		
Premenopausal	27 (60)	10 (50)
Postmenopausal	18 (40)	10 (50)
*ER status*		
Negative	0 (0)	0 (0)
Positive	45 (100)	20 (100)
*PR status*		
Negative	1 (2.2)	0 (0)
Positive	44 (97.8)	20 (100)
*HER2 overexpression*		
Negative	34 (75.5)	15 (75)
Positive	11 (24.5)	5 (25)
*Lymph node involvement*		
No	19 (45.2)	9 (45)
Yes	23 (54.8)	11 (55)
*Tumor size*		
T1: ≤2 cm	19 (42.2)	7 (35)
T2: >2 cm but ≤5 cm	21 (46.7)	10 (50)
T3: >5 cm	4 (8.9)	3 (15)
T4: any size with direct extension to chest wall and/or to skin	1 (2.2)	0 (0)
*Tumor grade*		
G1: Well differentiated	7 (15.6)	2 (10)
G2: Moderately differentiated	20 (44.4)	12 (60)
G3–G4: Poorly differentiated	18 (40)	6 (30)

### miRNA Profiling in Breast Cancer and Normal Adjacent Tissues

**Figure 1:**
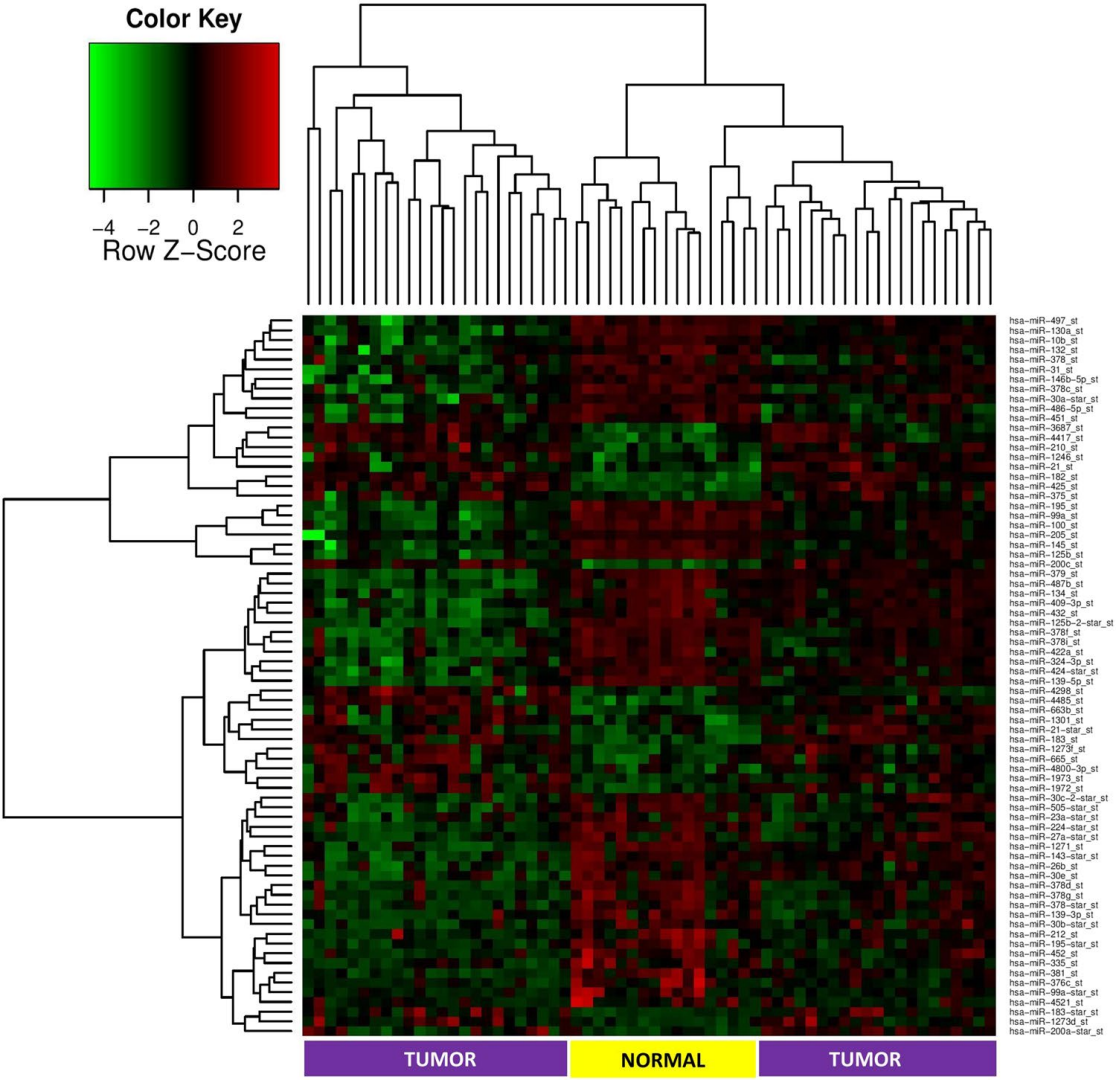
Heatmap of differentially expressed miRNA in tumor versus normal adjacent breast tissues taken from Lebanese patients. (Fold Change >2 and adjusted p-value < 0.05). Each row represents a differentially expressed miRNA and each column represents one of the 62 samples. The color key shown at the top left illustrates the relative expression level of a miRNA across all samples. A total of 74 miRNA were differentially expressed with separate clustering of tumor and normal adjacent tissues.

**Figure 2:**
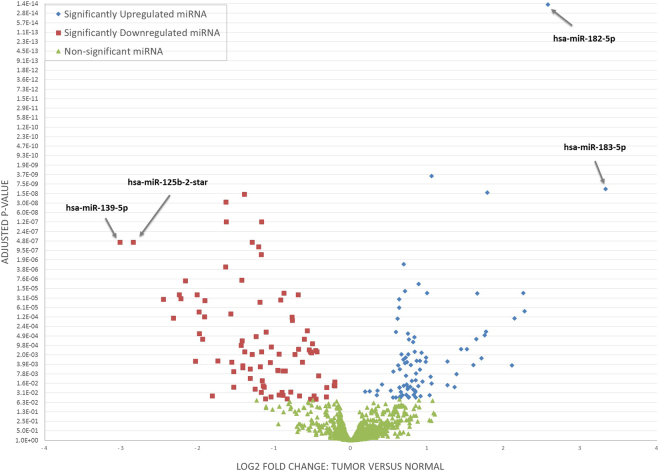
Volcano plot representing log2 fold change as a function of the adjusted p-value for miRNA expression in tumor versus normal adjacent breast tissues from Lebanese patients. The arrows highlight the upregulated and downregulated miRNA with the highest fold change.

miRNA microarray analysis in Lebanese tumor versus normal adjacent breast tissues revealed a total of 173 significant mature miRNAs (p < 0.05), of which 74 miRNAs were differentially expressed with fold change greater than 2 and p < 0.05 (Supplementary Table S1). Two-way hierarchical clustering of the 74 significant miRNAs resulted in separate clustering of tumor and normal adjacent tissues (Fig. 1). Of these 74 differentially expressed mature miRNAs, 23 miRNAs (31%) were up-regulated and 51 (69%) were downregulated. In addition, miR-183 and miR-182 were the most up-regulated miRNAs in tumor tissues with a fold change of greater than 5 times and miR-139-5p and miR-125b-2-star were the most down-regulated miRNAs with a fold change of greater than 7 times (Fig. 2).

### Validation of differentially expressed miRNA

**Figure 3:**
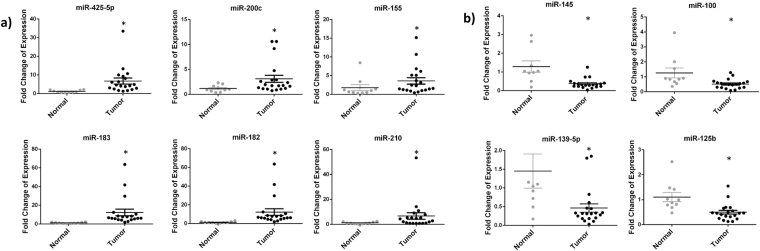
Validation of 6 significantly upregulated (**a**) and 4 significantly downregulated (**b**) miRNAs as per microarray results in tumor vs normal adjacent tissues. These dotplots show the fold change of miRNA expression in 20 tumor breast tissues normalized to the average of 10 normal adjacent breast tissues with RNU6B used as an endogenous control. The plot represents the mean with the standard error of mean as error bars. *Denotes p < 0.05 for tumor versus normal adjacent tissues using Wilcoxon’s signed-rank sum test.

In order to validate miRNA microarray results, quantitative real time-polymerase chain reaction (RT-qPCR) was performed to detect the levels of 4 randomly selected, significantly down-regulated miRNAs (miR-139-5p, miR-145, miR-100 and miR-125b) and 6 randomly selected, significantly upregulated miRNAs (miR-183, miR-182, miR-155, miR-210, miR-200c and miR-425-5p) on 20 tumor and 10 normal adjacent breast tissues (Table 1). As shown in Fig. 3, all of the 10 tested miRNAs showed significant dysregulation consistent with the microarray data using RNU6B as an endogenous control.

### mRNA Profiling in Breast Cancer and Normal Adjacent Tissues

**Figure 4:**
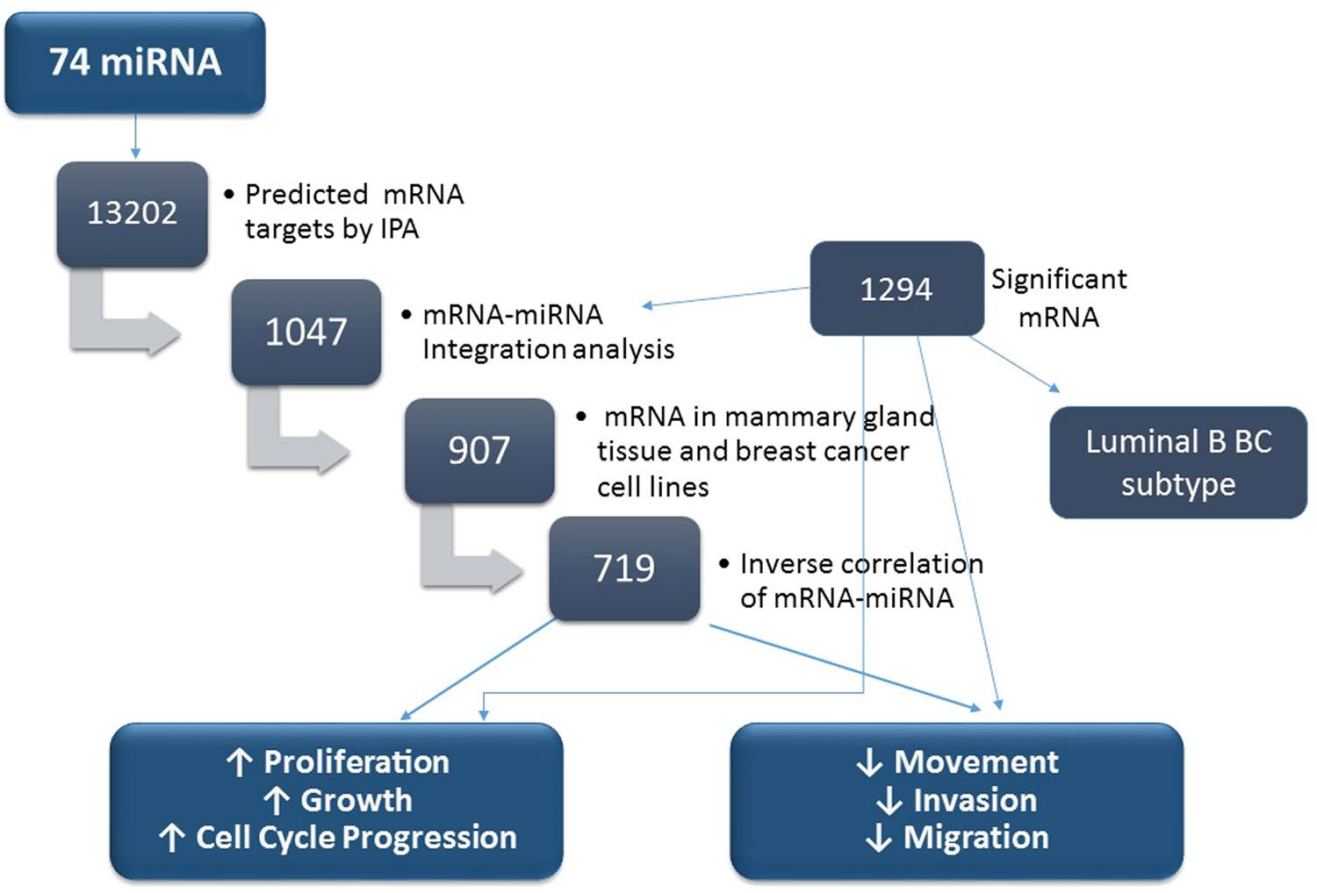
Filtration method for mRNA targets of the 74 differentially expressed miRNAs in tumor vs normal adjacent tissues from Lebanese patients using IPA. 13202 mRNA targets were predicted as potential targets of 74 dysregulated miRNA in Lebanese samples. The predicted miRNA targets decreased to 1047 mRNAs after miRNA-mRNA integration, and then to 907 mRNAs when specifying breast tissue. mRNAs were filtered to 719 when choosing an inverse correlation of mRNA and miRNA expression. 719 mRNA are involved in decreasing cell movement, invasion, and metastasis as well as increasing proliferation, growth and cell cycle progression.

**Figure 5:**
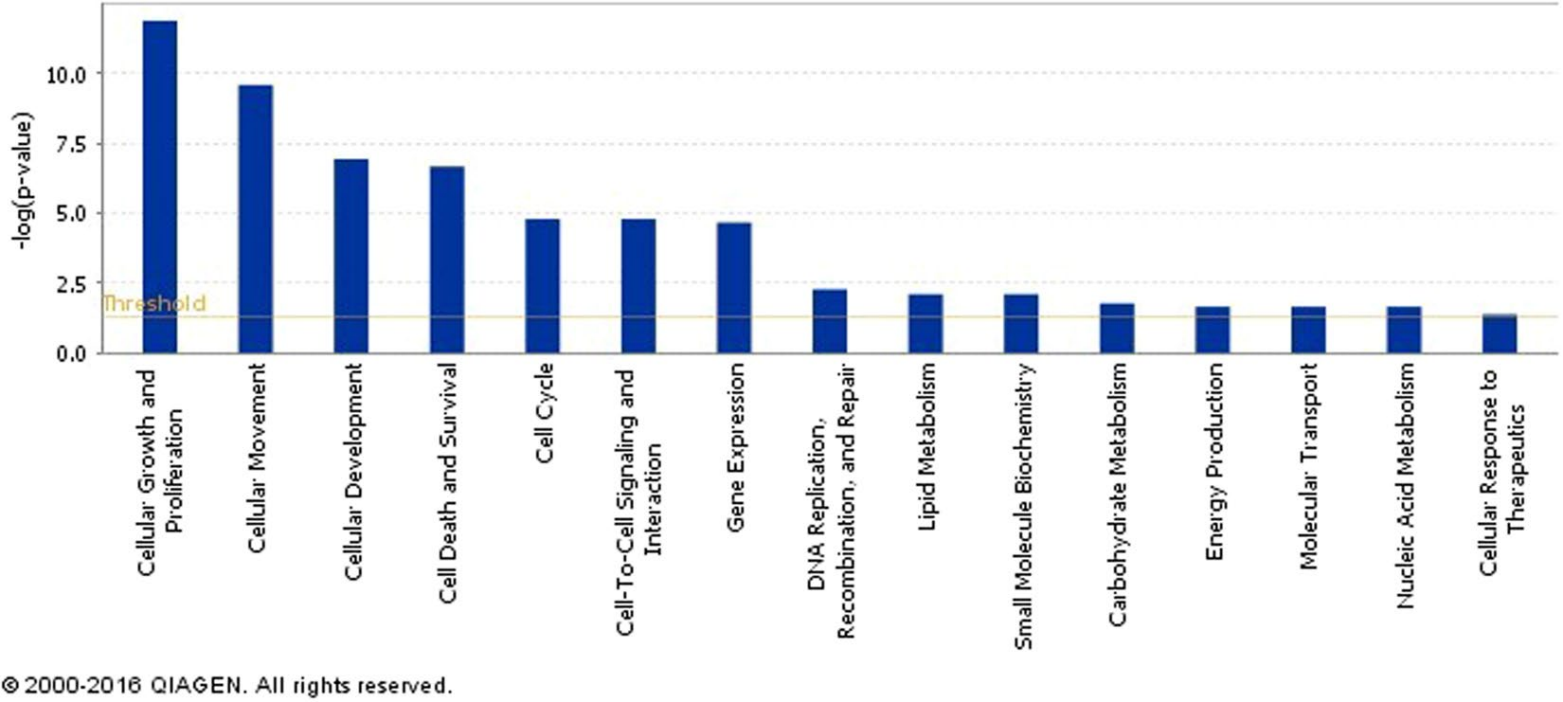
Ingenuity pathway analysis (IPA) of predicted cellular and molecular functions of all 1294 differentially expressed mRNA in tumor versus normal adjacent breast tissues. The most often predicted cellular function was cellular growth and proliferation. The p-value measures the probability of random association between mRNA and a related function.

In order to study the effect of miRNA on filtered targets more specific to breast cancer tissue, we performed mRNA microarray on RNA extracted from 11 of the formalin fixed paraffin embedded (FFPE) breast tissues (6 tumor and 5 normal adjacent tissues that were used in miRNA profiling) whose data served as a platform for miRNA-mRNA integration. All tumor samples were ER+, PR+, and HER2−, and from patients younger than 40 years old. mRNA microarray analysis have revealed 2251 significant transcripts representing 1294 genes (Fig. 4). Among the top upregulated mRNA were those produced by classical luminal genes such as *BMPR1B*, *ESR1*, *GATA3*, *KRT18, KRT8* and *FOXA1* ^7^. More specifically, the analysis revealed that the mRNA-based subtype of the Lebanese samples is luminal B as *SFRP1*, *KRT14*, *KRT5*, *EGFR*, *CDH3*, *MYC* and *FOXC1* were downregulated, while *MLPH*, *MAPT*, *ESR1*, *SLC39A6*, *CEP55*, *UBE2C*, *CDC6*, *PTTG1*, *CDC20* and *BIRC5* were upregulated in accordance to the differential expression of 50 genes used in the Prosigna breast cancer gene signature assay (PAM50)^8^. Like other luminal B tissues, our samples shared some gene patterns with luminal A such as upregulated ER genes *ESR1* and *FOXA1*, and some patterns with basal-like tissues such as increased expression of proliferation markers Ki-67 gene *MKI67*, survivin gene *BIRC5*, and cyclin B1 gene *CCND1* ^9^. Other dysregulated mRNA that can play important roles in tumor cell behavior as per MammaPrint breast cancer profile were the upregulated *DTL* and *RASSF7* mRNA whose proteins are involved in uncontrolled cell cycle and in microtubule cytoskeleton and spindle formation respectively^10–12^. As predicted by Ingenuity Pathway Analysis (IPA), all 1294 dysregulated mRNAs in the Lebanese breast cancer tissues were mainly involved in cancer and organismal injury and abnormalities in terms of diseases as well as cellular growth, proliferation and movement with respect to molecular and cellular functions (Fig. 5).

### miRNA-mRNA integration

**Figure 6:**
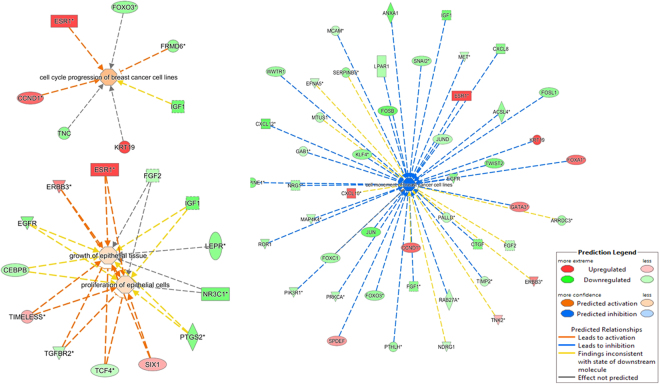
Significant functional networks of mRNA selected upon mRNA-miRNA integration analysis. Dysregulated mRNA in Lebanese population are mainly involved in cell movement, cell cycle progression, cell proliferation and growth as predicted by IPA.

IPA was utilized as an *in silico* tool to find the predicted and validated targets of the 74 differentially expressed miRNAs and to integrate the mRNA and miRNA microarray data. Primary results of IPA prior to miRNA-mRNA integration revealed 55 miRNAs with 13202 potential mRNAs targets (Fig. 4). Upon miRNA-mRNA integration, the predicted miRNA targets decreased to 1047 mRNAs and then to 907 mRNAs when selecting breast tissue in IPA (Fig. 4). Further filtering was performed to include 719 mRNAs involved in an inverse correlation of mRNA and miRNA expression (including over-expressed mRNA/under-expressed miRNA or under-expressed mRNA/over-expressed miRNA) (Fig. 4). The miRNAs with the highest number of targets in this analysis were miR-183 and miR-182, which also had the highest fold change (Supplementary Table S4). In order to determine the role of dysregulated miRNA in Lebanese breast cancer patients, we grouped the 719 mRNA according to biological processes that are based on QIAGEN’s Ingenuity Knowledge Base using Pathway Analysis tool in IPA. Most of the 719 predicted dysregulated mRNA targets turned out to be mainly involved in decreasing cellular movement, invasion and migration, increasing cell proliferation and growth, and increasing cell cycle progression as shown in Figs 4 and 6.

### Comparative Analysis of Lebanese and American miRNA Profiles

In order to compare Lebanese miRNA profile to another ethnic group, the miRNA sequencing data of 197 breast tumor and 87 normal samples of American samples were examined from TCGA (The Cancer Genome Atlas). Similar to Lebanese tumor samples, American tumor samples were from patients with IDC histotype, ER+/PR+ profile, and no distant metastasis. Analysis of TCGA data showed 396 significant miRNA in tumor versus normal tissues compared to 173 significant miRNA from Lebanese samples. Remarkably 81 miRNAs were significant solely in Lebanese patients, 60 of which were newly discovered miRNAs. The remaining 21 included: miR-422a, miR-31, miR-1246, miR-424-star, miR-30e, the miR-132/212 cluster, miR-362-3p, miR-663, miR-26b and miR-877. All miR-200 family members were significantly upregulated in American samples but only miR-200c was significantly upregulated in the Lebanese samples. miR-1228-star, miR-665, miR-146b-5p and miR-324-3p were dysregulated in opposite directions in the two patient sets. (Supplementary Table S3).

## Discussion

miRNA regulates at least 60% of protein coding genes^13^. Their dysregulation in cancer particularly in breast cancer highlights their importance in tumor development. This study focused on analyzing miRNA expression profile of tissues taken from Lebanese breast cancer patients, predicting the dysregulated miRNA function through mRNA-miRNA integration, and performing a comparative miRNA profile analysis with American specimens. Our study sample consisted mainly of patients diagnosed with the most common histotype and receptor profile in Lebanon (IDC and ER+/PR+). We identified 74 significant differentially expressed miRNAs in Lebanese patients’ tumor tissues, of which 43% of the miRNA were not previously studied in breast cancer and 23% were never reported in any cancer based on a literature review for involvement of each of the top 74 differentially expressed miRNAs in breast or other types of cancer (Supplementary Table S1). This suggests either a specific differential expression of miRNA in Lebanese breast cancer patients or the involvement of newly discovered miRNA that require further study (60% of those not involved in cancer and 43% of those not involved in breast cancer) (Supplementary Table S1). We have also identified 1294 significantly dysregulated mRNAs in tumor versus normal adjacent breast tissues that can classify the mRNA-based subtype of Lebanese samples as luminal B. Luminal B subtype is the more aggressive type of ER+ breast cancer that is associated with a risk of early relapse because luminal B tumors have higher expression of proliferation/cell cycle-related genes as compared to luminal A tumors^9,14^. Using IPA, the predited role of 1294 mRNAs is increasing cellular growth, proliferation and decreasing cell movement which was similar to the predicted role of 719 mRNAs filtrated as targets for the 74 differentially expreressed miRNAs.

### miRNA role in increasing cell proliferation and cell cycle progression

**Figure 7:**
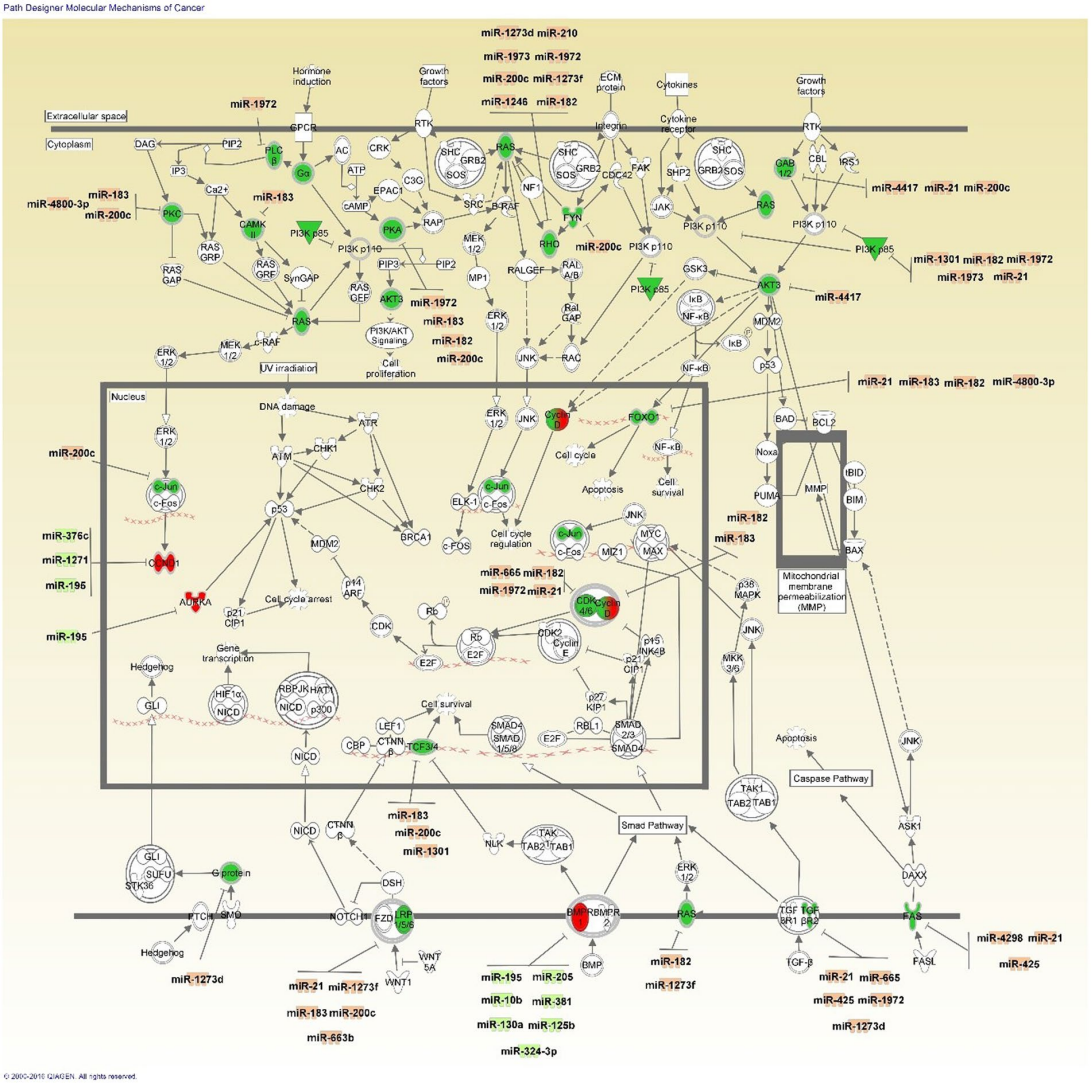
Canonical pathway of molecular mechanisms of cancer from IPA showing dysregulated mRNA targets along with their potential differentially expressed miRNA regulators found in Lebanese samples. Red denotes increased expression and green decreased expression. Note: CCND1 is upregulated while CCND2 is downregulated but both are represented as Cyclin D.

mRNA-miRNA integration and anticorrelated analyses of miRNA and mRNA expression have shown that dysregulated miRNA could be involved in tumor cell proliferation and growth as well as cell cycle progression (Supplementary Table S4). The under-expression of miR-497, 376c and 1271 in Lebanese breast cancer tissues could explain the observed upregulation of *CCND1* that encodes for cyclin D1, promotor of cell cycle progression through inhibition of tumor suppressor Rb (Fig. 7). Cyclin D1 over-expression has been associated with poor prognosis of women with ER+ breast cancer^15,16^. However, *CCND1* gene amplification is not the only reason for its over-expression as it might also be controlled by more than one epigenetic mechanism^17^. The upregulated miR-183 in our samples was predicted to be responsible for the decrease in expression of the *BTG1* mRNA whose protein is involved in cell cycle arrest and apoptosis in breast cancer cells^18^.

Another molecule related to cell proliferation that was over-expressed in our data and suggested as a target of downregulated miR-376c is *AURKA* (Fig. 7). Its protein is a key regulator of chromosome segregation and cytokinesis that is correlated with distant metastases in ER+ breast cancer^19^. Moreover, the over-expression of miR-183 and miR-21 in Lebanese breast cancer tissues is consistent with downregulation of two important tumor suppressor predicted targets: *AKAP12* whose protein regulates cellular adhesion dynamics by controlling cytoskeletal architecture, cell migration, and mitogenic signaling^20^; and *LATS2* whose protein causes cell cycle arrest. *LATS2* mRNA downregulation has been correlated with aggressive breast cancer and has been attributed to hypermethylated promotors; however, miRNA could be another mode of its regulation^21^.

*PI3KR1* encodes for phosphoinositide-3-kinase regulatory subunit 1 (p85α), which has a tumor suppressive role, and was downregulated in our samples possibly by the detected upregulated miR-21, 1972, 1301, 1973 and 182 (Fig. 7). Gene mutations of this gene are an uncommon cause of its under-expression in breast cancer, so the gene could be possibly under miRNA regulation^22^. *PI3KR1* under-expression is also associated with other PI3K-Akt pathway molecules such as *AKT3* downregulation, which could be due to the over-expression of miR-4417^23^. Additional molecules with anti-proliferative and pro-apoptotic effect whose mRNA were suppressed in our samples are FOXO1 (Fig. 7) and FOXO3^24–26^. Their mRNA expression is a potential target of miR-21/183/182/4800-3p and miR-182/21 respectively. Other downregulated members of the forkhead box transcription factor family are FOXC1 and FOXP1, whose mRNA are possible targets of the over-expressed miR-1301 and miR-183/200c respectively. Loss of FOXC1 expression is an early event during breast cancer progression and could be inhibited by methylation^27^ or another epigenetic change such as miRNA dysregulation. The downregulation of *DICER1* mRNA in our samples is a potential effect of the detected miR-425 over-expression. Dysregulated DICER1 in breast cancer could explain the global decrease in miRNA expression^28^, consistent with our results.

### miRNA role in decreasing migration and invasion

In addition, the dysregulated miRNA in Lebanese breast tumor samples were predicted to help inhibit migration and invasion. This can be explained by the fact that the breast cancer samples were taken from early stage patients without distant metastases. The mRNA of important genes in the epithelial mesenchymal transition required for invasion and migration are downregulated including *SNAI2*, *TWIST1*, *TWIST2*, and *ZEB2* and are likely under the control of miRNA that we found were upregulated; miR-182/1301, miR-1972, miR-4417, and miR-183/182/200c/3687 respectively^29–31^. As such, these latter dysregulated miRNA could have a different expression in metastatic breast cancer. This is in accordance with a previous study whereby miRNA dysregulation differed between invasive and less-invasive breast cancer cell lines^32^.

A comparative miRNA profile analysis between Lebanese and American samples revealed that most of the dysregulated miRNA in Lebanese patients were similar to that of American patients. Nevertheless, 81 miRNA were exclusively dysregulated in the Lebanese specimens. Among them is the upregulated miR-1246 which was found to be selectively released in serum of patients with early breast cancer^33,34^. miR-31 and the miR-212/132 cluster, both known for their anti-metastatic roles, were exclusively downregulated in Lebanese samples and which could make breast cancer in this population more prone to metastasis^35,36^ and thus explain the disparity in outcomes between younger women in Lebanon compared to America. Interestingly, many of the miRNA exclusively dysregulated in Lebanese patients (miR-31, miR-362-3p and miR-663) and in the TCGA data (miR-329, miR-22, miR-373, miR-320a, miR-34b, miR-196a, miR-149, miR-203) are controlled by promoter hypermethylation^37–42^. This suggests methylation studies as another potentially fruitful line of inquiry for explaining breast cancer outcomes in Lebanon. Other miRNA (miR-196a, miR-185 and miR-206) exclusive to TCGA data were shown to have sequence variance in their precursors that may affect their processing^42,43^. This suggests the possibility that miRNA expression could be differentially expressed across ethnicities, possibly due to an epigenetic mechanism affected by patient lifestyle and/or environmental exposures. Our study adds credence to the idea of population-specific miRNA variations which was previously reported by Rawlings-Goss *et al*.^44^. In this study, population-specific miRNA variations were identified in a large genetic study performed on 69 unrelated individuals from 14 global populations including Europeans, Asians, and Africans. miR-1908, miR-520h and miR-196a, which were among the 7 population differentially expressed miRNAs^44^, were differentially expressed between Lebanese and American population in our study as well. In addition, miR-1288-star which was upregulated in our population but under-expressed in TCGA data, was found to be significantly upregulated in young women similarly to miR-3196, which has also been found to be significantly over-expressed only in Lebanese populations^45^. When comparing the age of our study population to that of TCGA, we had a larger number of young patients which may explain the upregulation of miR-1228-star and miR-3196 in our data. Another comparative analysis of miRNA expression was performed taking into consideration tissue samples from Lebanese and American patients who were below 40 years of age. We found that 146 miRNA were differentially expressed in both Lebanese and American young patients. 27 miRNAs were exclusively dysregulated in American samples and 145 miRNAs were only differentially expressed in Lebanese patients. Most of 145 miRNA are not well studied and their functions are yet unknown. Interestingly, Lebanese samples have a significant upregulation (more than 2 folds) in two members of miR-200 family (miR-141 and miR-200b) as well as a significant downregulation (more than 2 folds) in miR-138, miR-224, miR-148a and miR-223. These could be used later as diagnostic and prognostic biomarkers. As a matter of fact, high level of circulating miR-141 was associated with brain metastasis from breast cancer^46^, and in triple-negative breast cancer cells, elevated expression promoted migration and invasion through the activation of the FAK and PI3K/AKT signaling pathways^47^. In addition, high expression of miR-141 and low expression of miR-223, 224 and miR-148a were associated with chemoresistance^48–51^. Moreover, low expression of miR-148a has been reported in basal and luminal B subtype primary tumors, and it was associated with poor prognosis of breast cancer patients^52^.

Our comparative analysis has some limitations including the higher frequency of young patients (less than 40 years old) and the smaller sample size of our study as compared to TCGA data as well as the difference in platforms for both sets of data. TCGA data used a sequencing platform that tends to detect more differential expression than our microarray platform; thus explaining the high number of miRNA dysregulated in the TCGA data. Another limitation related to integration analysis is the exclusion of predicted mRNA targeted by miRNA at regions other than 3′ untranslated region since IPA uses only TargetScan as an *in silico* prediction tool.

In conclusion, mRNA-miRNA integration analysis of early breast cancer revealed a potential role of miRNA in increasing cellular proliferation and progression, and decreasing invasion and migration. Moreover, although most of the miRNA dysregulated in Lebanese breast cancer patients are similar to those dysregulated in American patients, differences in miRNA expression exist and could be attributed either to the patients’ age at diagnosis or to ethnic variation in miRNA epigenetic regulation and sequence variation of pre-miRNA. Our data provide a basis for further genetic/epigenetic investigations to identify ethnic specific miRNA, to uncover the role of newly identified miRNA and to comprehend their implications in early stage breast cancer.

## Methods

### Breast Cancer Tissue Specimens

The research protocol was approved by the Institutional Review Board (IRB) at the American University of Beirut (IRB ID#: IM.RN.02) and all patients who participated in the study signed a written informed consent form. All methods were conducted in accordance with approved guidelines and regulations. FFPE sections from invasive ductal carcinoma specimens (N = 45) and normal adjacent tissues (N = 17) were obtained at the American University of Beirut Medical Center in Lebanon from samples collected between 1997 and 2012. Clinical and pathological data including age at diagnosis, menopausal status, tumor pathology, stage, grade, ER status, PR status, and HER2 over-expression were available for all samples included in this study.

### Total RNA Extraction

Total RNA was extracted from 45 tumor and 17 normal adjacent breast tissues in accordance with the RecoverAll™ Total Nucleic Acid Isolation Kit protocol for FFPE samples (Ambion, USA). RNA concentration and quality was assessed using Nanodrop ND1000.

### miRNA Microarray and Data Analysis

Sixty-two RNA samples (150 ng in 8 µl) were labeled using the FlashTag™ Biotin HSR Labeling Kit in accordance with the manufacturer’s instructions. RNA was first poly-A tailed followed by ligation with a biotinylated signal molecule and then hybridized for 18 hrs at 48 °C to the GeneChip miRNA 3.0 Array (Affymetrix Inc., Santa Clara, CA, USA). The miRNA microarray contains probes designed for all miRNA in miRBase Release 17 with 1,733 human mature and 1,658 human pre-miRNA probe sets. Washing and staining were performed according to standard Affymetrix protocols and the arrays were scanned by an Affymetrix GCS 3000 7 G Scanner.

Data were analyzed within the R statistical environment using Bioconductor (http://www.bioconductor.org) packages. miRNA from *Homo sapiens* were extracted from the array. Data were normalized with the Robust Multiarray Average algorithm^53^. To detect differentially expressed miRNA in tumor and normal adjacent breast tissue samples, the R package Limma^54^ was used to fit a linear model to normalized expression data for each probe. False Discovery Rates (FDR) were estimated using the Benjamini-Hochberg method^55^. The normalized expression matrix was used in clustering analysis to see whether the sample phenotypes correlated with miRNA expression. Significant miRNA in tumor versus normal adjacent tissues with an adjusted p-value less than 0.05 were considered differentially expressed if their fold change was greater than 2.

### Validation of miRNA expression by RT-qPCR

Twenty tumor and ten normal adjacent breast tissues were randomly selected to validate miRNA expression. Reverse transcription of ten nanograms of total RNA was performed using the TaqMan® MicroRNA Reverse Transcription Kit (Applied Biosystems, USA) according to the manufacturer’s instructions. Small nuclear RNA RNU6B, hsa-miR-183, hsa-miR-182, hsa-miR-210, hsa-miR-200c, hsa-miR-125b, hsa-miR-100, hsa-miR-425-5p, hsa-miR-139-5p, hsa-miR-145 and hsa-miR-155 primers and probes (Assay ID: 001093, 002269, 002334, 000512, 002300, 000449, 000437, 001516, 002289, 002278 and 000479) were purchased as part of the TaqMan® microRNA Assays Kit (Applied Biosystems, USA) with validated efficiency. Following cDNA synthesis, RT-qPCR was performed using BioRad CFX96 Real Time System, C1000 Thermal Cycler (Germany). Reactions using 2x TaqMan® Universal Master Mix with no Amperase Uracil N-glycosylase (Applied Biosystems, USA) were performed in duplicate for each miRNA probe. The cycling conditions were 95 °C for 10 min and 40 cycles of 95 °C for 15 seconds and an annealing temperature of 60 °C for 60 seconds. Using the ΔΔCq equation, relative expression of the experimental miRNA was determined in the tumor samples compared to normal adjacent tissues using RNU6B as an endogenous control. Normalization of tumor tissues was based on normal adjacent tissues present on the RT-qPCR plate to ensure inter-run calibration. Statistical analyses were performed using SPSS software package version 18. Wilcoxon’s signed rank sum test was used to compare the miRNA expression in the tumor versus the normal adjacent tissues. A p-value < 0.05 was considered statistically significant.

### mRNA Microarray and Data Analysis

Eleven RNA samples (100 ng in 5 µl) [6 tumor and 5 normal adjacent breast tissues randomly selected from the young Lebanese breast cancer patients] were first amplified using the SensationPlus™ FFPE Amplification Kit (Affymetrix Inc., Santa Clara, CA, USA) in accordance with manufacturer’s instructions. The senseRNA was then converted into labeled cDNA using the SensationPlus™ 3′ IVT Labeling Kit. The cDNA was hybridized for 18 hrs at 48 °C to the GeneChip® Human Genome U133 Plus 2.0 Array (Affymetrix Inc., Santa Clara, CA, USA). The mRNA microarray contains 1,300,000 probe designed for over 47,000 transcripts and variants that represent approximately 39,000 of the best characterized human genes. Washing and staining were performed according to standard Affymetrix protocols and the arrays were scanned by an Affymetrix GCS 3000 7 G Scanner. Sample data were analyzed within the R statistical environment similar to the miRNA microarray. Significant mRNA in tumor versus normal adjacent tissues were considered differentially expressed if their adjusted p-value is less than 0.05.

### *In silico* tools for mRNA-miRNA integration

Data was analyzed using QIAGEN’s Ingenuity® Pathway Analysis (IPA®, QIAGEN Redwood City, www.qiagen.com/ingenuity). IPA Downstream Effects Analysis aims to predict biological functions depending on validated literature compiled in the Ingenuity® Knowledge Base in comparison to observed gene expression changes in the experimental dataset. A p-value representing the probability that each function or disease is randomly associated with each mRNA was calculated using Fisher’s exact test. miRNA-mRNA integration was performed using IPA miRNA Target Filter based on TargetScan Human, Tarbase, miRecords, Ingenuity Expert Findings and Ingenuity ExpertAssist Findings.

### TCGA Data Analysis

TCGA breast invasive carcinoma miRNASeq data were downloaded from TCGA (The Cancer Genome Atlas, https://tcga-data.nci.nih.gov/tcga/), and matched our samples with IDC histotype, ER+/PR+ profile, and no distant metastasis. In total, 197 breast tumor and 87 normal samples were included in the analysis taken mainly from Caucasian patients. Limma was used to compare miRNA expression differences between tumor and normal samples, with FDR < 0.05 was considered statistically significant.

### Data availability

The datasets generated from microarray and analysed during the current study are available on the Geobrowser (to be assigned a number when published).

## Electronic supplementary material

Supplementary Table S1.
